# Alternate Entropy Computations by Applying Recurrence Matrix Masking

**DOI:** 10.3390/e24010016

**Published:** 2021-12-23

**Authors:** Charles L. Webber

**Affiliations:** Department of Cell and Molecular Physiology, Health Sciences Campus, Loyola University Chicago, 2160 South First Avenue, Maywood, IL 60153, USA; cwebber@luc.edu; Tel.: +1-708-638-7497

**Keywords:** nonlinear dynamics, recurrence quantifications, line entropy, recurrence matrix masking

## Abstract

In practicality, recurrence analyses of dynamical systems can only process short sections of signals that may be infinitely long. By necessity, the recurrence plot and its quantifications are constrained within a truncated triangle that clips the signals at its borders. Recurrence variables defined within these confining borders can be influenced more or less by truncation effects depending upon the system under evaluation. In this study, the question being asked is what if the boundary borders were tilted, what would be the effect on all recurrence variables? This question was prompted by the observation that line entropy values are maximized for highly periodic systems in which the infinitely long line elements are truncated to different unique lengths. However, by redefining the recurrence plot area to a 45-degree tilted box within the triangular area, the diagonal lines would consequently be truncated to identical lengths. Such masking would minimize the line entropy to 0.000 bits/bin. However, what new truncation influences would be imposed on the other recurrence variables? This question is examined by comparing recurrence variables computed with the triangular recurrence area versus boxed recurrence area. Examples include the logistic equation (mathematical series), the Dow Jones Industrial Average over a decade (real-word data), and a square wave pulse (toy series). Good agreement among the variables in terms of timing and amplitude was found for most, but not all variables. These important results are discussed.

## 1. Introduction

As an extension of recurrence plots [1], recurrence quantification analysis (RQA) was introduced by Zbilut and Webber [2,3] almost three decades ago and Marwan et al. [4] two decades ago. These quantifications include eight recurrence variables extracted from recurrence plots [5] which have proven to have utility in general-purpose data analyses for linear and nonlinear systems alike [6]. The most challenging aspect of recurrence analyses is the setting of the multiple recurrence parameters [7]. What may be less appreciated is the effect border truncations have on all recurrence variables.

The fundamental feature of recurrence plots, which distinguishes deterministic from stochastic systems, is the presence of diagonal line structures. Since the traditional recurrence plot is symmetrical on either side of the line of identity (LOI), only the upper-triangular half of the plot is utilized. The ubiquitous LOI is excluded. Three recurrence quantification variables are directly derived from these diagonals. First, percent determinism (DETERM) is defined as the ratio of points forming diagonal line structures to the total number of recurrent points. Second, the diagonal maximum (DMAX) is defined as the integer number of points constructing the longest diagonal line. Third, information or line entropy (ENT) is defined as the Shannon entropy [8] of the histogram distribution of all diagonal line lengths within the triangular recurrence window. Because long diagonal lines are necessarily truncated at the borders of the recurrence plot, the question arises how much the truncation effect influences the accuracy of DETERM, DMAX and ENT computations, not to mention the remaining recurrence variables. This is the same question formerly asked by Kraemer and Marwan [9].

This paper will focus on the effect recurrence borders have on the computation of line structures and other features of the recurrence plot. Two types of borders are examined: traditional triangular recurrence borders and novel tilted box recurrence borders. The latter represents a smaller area masked off within the larger triangular area. System studied include a mathematical system, a real-world financial system, and a contrived toy system to clearly illustrate time and amplitude shifts in all compared variables.

## 2. Disparity between Recurrence Line Entropy Values and Lyapunov Exponents

When this author [10] was studying the logistic equation by recurrence analysis, it was noted that when the equation was in period 1, period 2, period 4, etc., periodic modes, the line entropy values were maximized when the Lyapunov exponents were low. This counterintuitive relationship is easily explained by the differing lengths of diagonal lines which were truncated by the triangular borders into unique lengths. Censi et al. [11] also realized this effect and introduced a correction by assigning all diagonals to the same length as the central LOI. This method effectively minimized the line entropy values but carried with it the assumption that all lines are of equal length. Eroglu et al. [12] took a different approach using weighted recurrence plots. Entropic measures were redefined according to the distribution of return times, not line lengths. Indeed, Shannon entropy values became positively correlated with the largest Lyapunov exponent. More sophisticated approaches for quantifying recurrence entropy are based on matrix microstates [13] and categorical time series [14]. Finally, Kraemer and Marwan [9] described another method similar to the one introduced in this paper. That is they recomputed entropy by masking the recurrence plot such that the square recurrence matrix was windowed within a diamond pattern overlaying the traditional plot. Thus, for fully periodic systems, all diagonal lines were truncated to identical lengths, minimizing line entropy values to 0.000 bits/bin.

Because the auto-recurrence plot is symmetrical, in this paper, only the upper triangle was masked with the best fitting square (not half-diamond or rectangle as in the Kraemer-Marwan case). The box-masking will be fully described. The masking gives low entropy values for periodic systems and high entropy values for chaotic systems. It was necessary to compare these boxed entropy values against the traditional triangular entropy values for several different cases and situations. Indeed, performance testing was accomplished for all eight recurrence variables. The necessary comparisons include amplitude and timing characteristics of the recurrence variables for both triangular and boxed recurrences.

## 3. Redefinition of Recurrence Plot Boundary Conditions

**Figure 1:**
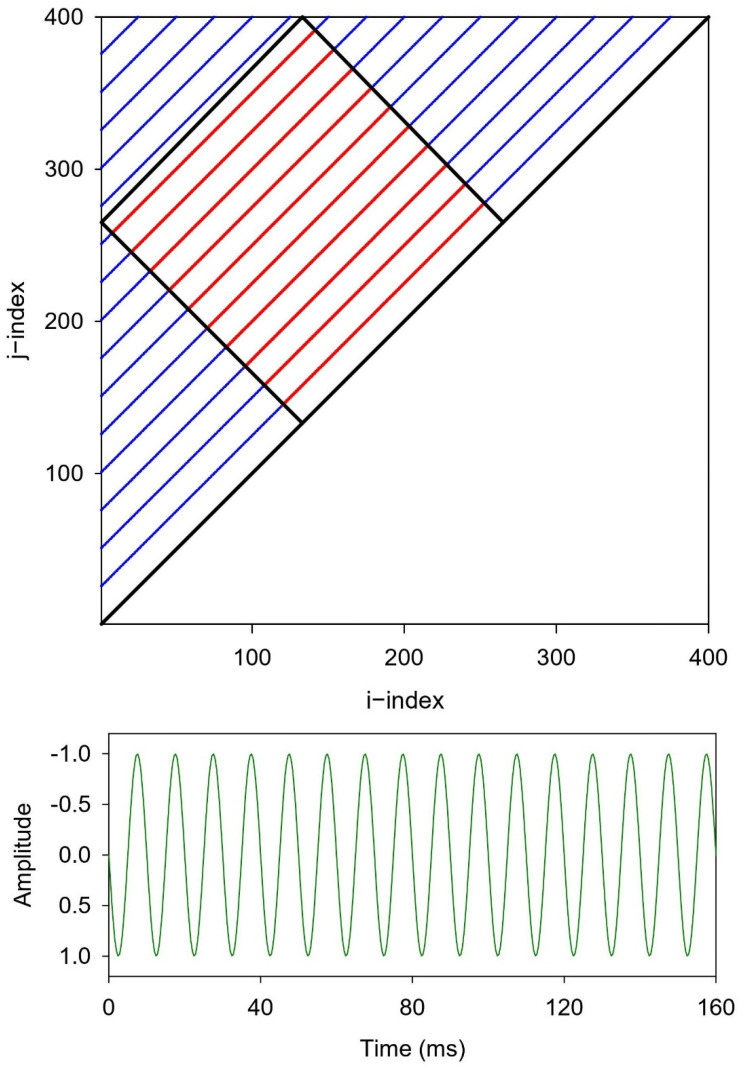
Recurrence plot of a sinewave with no added noise. The 16 noise-free sinewave cycles represent a 100 Hz sinewave digitized at 2500 Hz (25 points/cycle). The boxed recurrences (red lines) are constrained within the tilted box. The triangular recurrences (blue lines + red lines) are constrained with the upper half of the recurrence plot (above the LOI). Parameter settings: DELAY = 1; EMBED = 2; NORM = Euclid; WINDOW = 400; RESCALE = max distance; RADIUS = 1%; LINE = 2.

The boxed recurrence masking can be best be illustrated graphically. Figure 1 presents the 400-point recurrence plot of a simple repeating sinewave consisting of 16 cycles or waves as computed by traditional recurrence programs RQD and RQC, and new boxed programs RQDB and RQDC (see Appendix A). Because all 16 sinewaves are identical across the time series (green), 15 border-to-border lines excluding the LOI (black) are inscribed within the triangular recurrence plot (blue and red). Each one of these deterministic lines is truncated to a unique (different) length spanning from the west border to the north border. That is, the further the line is removed from the LOI, the shorter that line is. This has ramifications for line entropy calculations as described below.

Another approach to this sinewave truncation issue is to fashion a tilted box within the recurrence triangle parallel to the LOI (45-degree, clockwise tilt) as is illustrated by the black square (Figure 1). All recurrent points within this smaller boxed area (red) are simply shorter parts of the longer lines within the larger triangular area (blue). The advantage of this system is that the 10 diagonal line structures within the box are each truncated to identical lengths.

**Figure 2:**
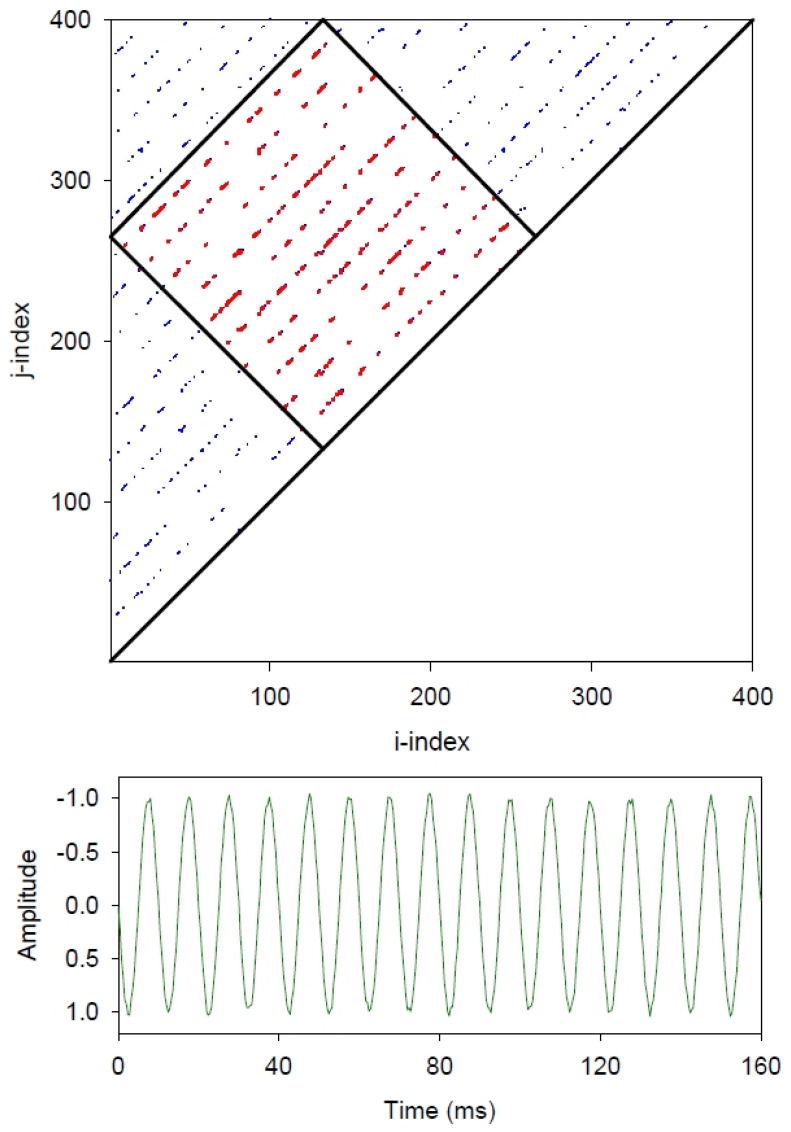
Recurrence plot of a sinewave with 5% added noise. The 16 noise-free sinewave cycles represent a 100 Hz sinewave digitized at 2500 Hz (25 points/cycle). The boxed recurrences (red lines) are constrained within the tilted box. The triangular recurrences (blue lines + red lines) are constrained with the upper half of the recurrence plot (above the LOI). DELAY = 1; EMBED = 2; NORM = Euclid; WINDOW = 400; RESCALE = max distance; RADIUS = 1%; LINE = 2.

Then, when 5% random noise is added to the pure sinewave, the long diagonal line structures are chopped up into shorter segments as shown in Figure 2. In this case, the noise mitigates the influence of the truncation effects of the triangular borders insofar that the line structures within the tilted box and triangle appear very similar. Are they in reality? By the way, the embedding dimension was set to 2 for both the pure sinewave with a radius of 1% of the maximum distance. Using these parameter settings there was no false thickening of the diagonal lines as discussed by Thiel et al. [15]. That is, the selected radius was high enough to include the noisy points, but not so high as to thicken the lines.

## 4. Tilted Box Boundaries and Line Entropy Modifications

**Figure 3:**
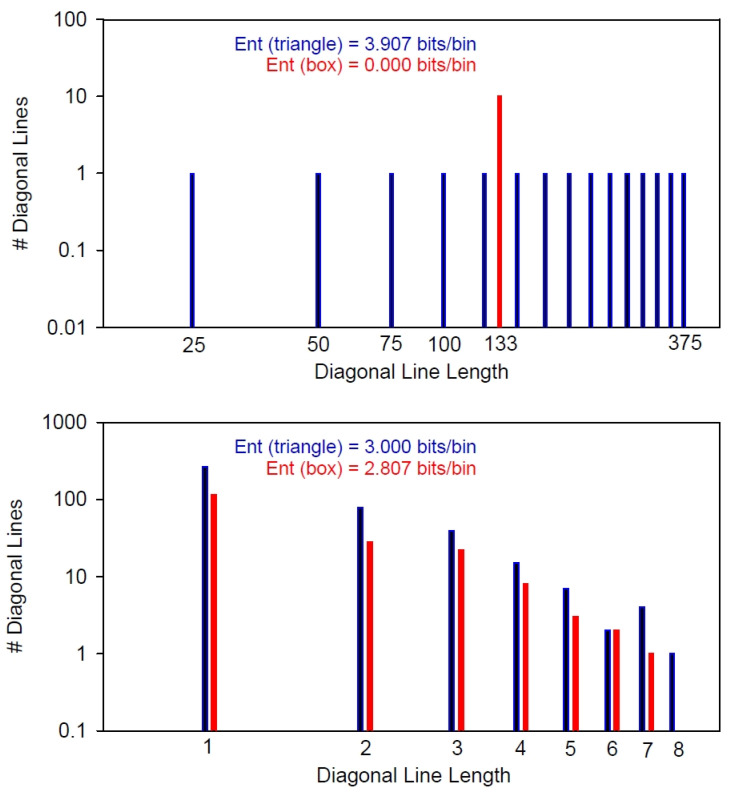
Histograms of diagonal line length distributions for noise-free sinewave (upper panel) and sinewave with 5% added noise (lower panel). Diagonal line lengths and their counts are shown for triangular recurrences (blue) versus boxed recurrences (red). Corresponding, color-coded entropy values are also given. Note that entropy values in the top panel are very different, but entropy values in the lower panel are very similar.

Figure 3 is key to understanding the differences of the boxed entropies over the triangular entropies computed from the histogram distribution of diagonal lines within the recurrence plot. The upper histogram is the distribution of diagonal lines within the noise-free sinewave (Figure 1) and the lower histogram is the distribution of diagonal lines within the sinewave with 5% added noise (Figure 2). Note that both horizontal and vertical scales are base-10 logarithmic. In the first case (blue lines), all 15 line lengths are of differing lengths (375, 350, 325, 300,…, 100, 75, 50 25 points) and there is but one unique line length of each. However, for the second case (red line), the 10 diagonal lines are all of identical length (133 points) and fall into a single histogram bin. Consequently, the triangular entropy computes as 3.907 bits/bin, whereas the box entropy computes as 0.000 bits/bin (see below).

Computation of line entropy follows the generalized formula used for Shannon information entropy [8] as shown in Equation (1). The entropy value is maximized when each non-zero bin has the same number of counts (identical probabilities) as computed by Equation (2). Conversely, the entropy value is minimized if bin counts are restricted to a single bin. In this case, the entropy falls out as 0.000 bits/bin as given by Equation (3). So from Figure 3, where 15 bins are each filled with the count of one (blue) the entropy maximized (−log_2_(1/15) = 3.907 bits/bin). However, for the 10 boxed lines (red), each line is 133 units long and the entropy is minimized (−log_2_(1/1) = 0.000 bits/bin).

ENT_gen_ = −Σ(P_bin_)log_2_(P_bin_) (1)

ENT_max_ = −log_2_(1/N_bin_) (2)

ENT_min_ = −log_2_(1/1) = 0.000 bits/bin (3)

So for noise-free sinewaves, at least, the computed values for line entropy range from maximal entropy (triangular recurrence area) to minimal entropy (tiled box recurrence area). It is the tilted box masking that makes all the difference. Both entropy values are mathematically correct, but the boxed entropy makes more sense with respect to the complexity of the signal. This is not globally true for all situations and signals.

With the jostling of the pure sinewave with 5% random noise, the long diagonal lines both within the boxed area and within the triangular area get parceled (chopped up) into shorter segments (Figure 2). Quantitatively, the distributions of these shortened line segments are very similar (Figure 3), which is why the information entropy values for both the triangular recurrences and boxed recurrences are likewise very similar (3.000 versus 2.807 bits/bin, respectively). Since noise is ubiquitous in real-world systems, possibly the two methods of entropy computations are not that much different after all. However, such a (good) conclusion has yet to be verified using experimental data.

## 5. Boundary Details of the Tilted Recurrence Box within the Recurrence Triangle

**Figure 4:**
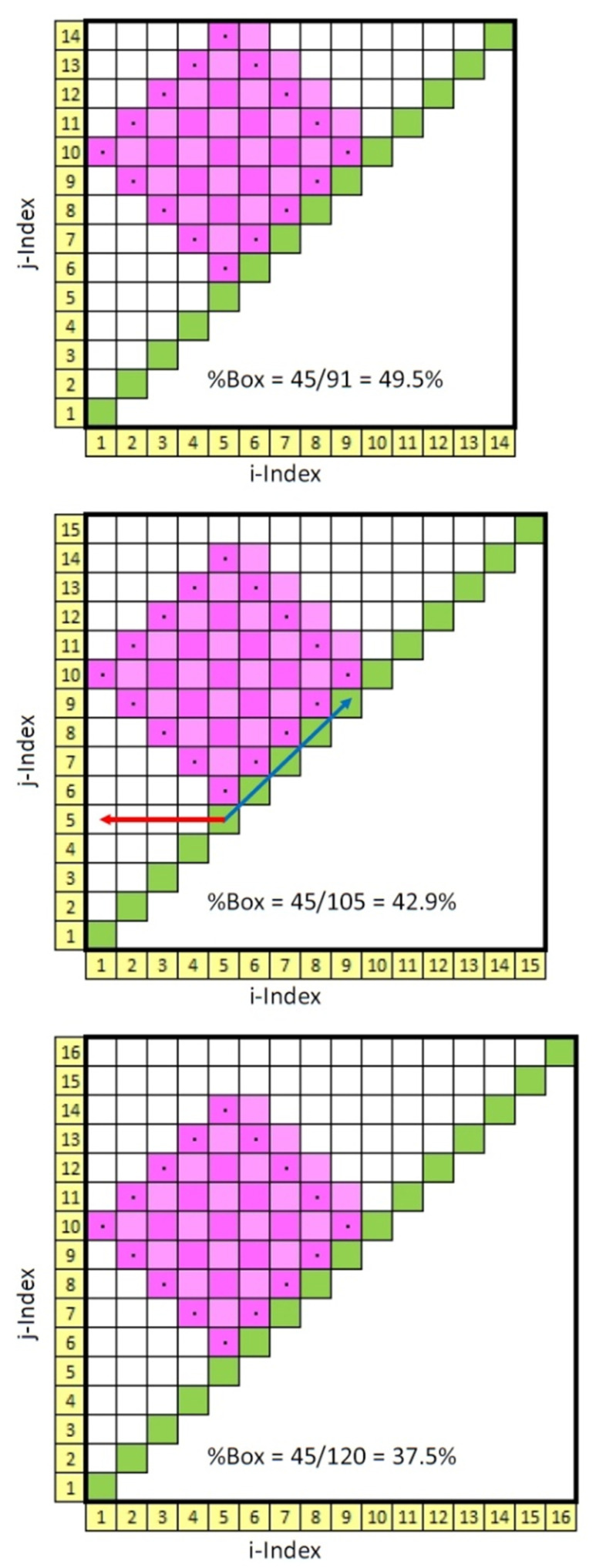
Tilted recurrence box of 5 × 5 units (dark and light pink pixels) fits within 3 recurrence matrices of 14 × 14 units (top), 15 × 15 units (middle), and 16 × 16 units (bottom). %Box is the ratio of the box areas to the triangle areas (excluding the green LOI) which progressively decreases as the size of the recurrence matrix increases. The blue arrow designates the middle third of five input points (e.g., P5–P9) from which the mean and standard deviation are computed. The red arrow indicates the left shift in the boxed recurrence variables by 4 points (P5–P1) for alignment with the triangular recurrence variables.

The edges of the recurrence box are not smooth, and the shape of the box is not perfectly square as implied by Figure 1 and Figure 2. For example, let us construct the largest 5 × 5 tilted square within a 14 × 14 recurrence matrix, half of which is shown in the upper panel of Figure 4. Here, the edges of the tilted square box are marked with black dots. As can be seen, each side of the box is 5 units in length. However, the area of this square is not 25 square units (dark pink pixels) but must include all gap spaces as well (light pink pixels). Secondly, the length of the diagonal must all be the same unit length (5 pixels here) increasing the box area to 45 square units (Equation (4)). Thirdly, the area of the triangle includes the box area plus all other empty pixels (white pixels) excluding the LOI (green pixels) (Equation (5)). Taking the ratio of box to rectangle areas computes the percentage of the recurrence plot occupied by the tilted box (Equation (6)).

Area_box_ = Side_box_ · Side_box_ + Side_box_ · (Side_box_ − 1) (4)

Area_triangle_ = (Side_triangle_ · Side_triangle_ − Side_triangle_)/2 (5)

%Box = 100 · Area_box_/Area_triangle_ (6)

Now, if the 14 × 14 recurrence matrix size is increased to 15 × 15 square units or even to 16 × 16 square units, the shape and area of the tilted recurrence box as defined above remain the same. This is proven graphically in panels 2 and 3, respectively, in Figure 4. At the same time, however, the area of the triangles increases as more empty pixels are added. Consequently, the ratios of box area to triangle area (%Box) must necessarily decrease. For this trio of pairs (Figure 4) the ratios decrease from 49.5% to 42.9% to 37.5% as indicated. However, by incrementing the size of the recurrence matrix up by one step to 17 × 17 square units, the legal tilted-box size likewise increases from 5 × 5 to 6 × 6 (not shown).

**Figure 5:**
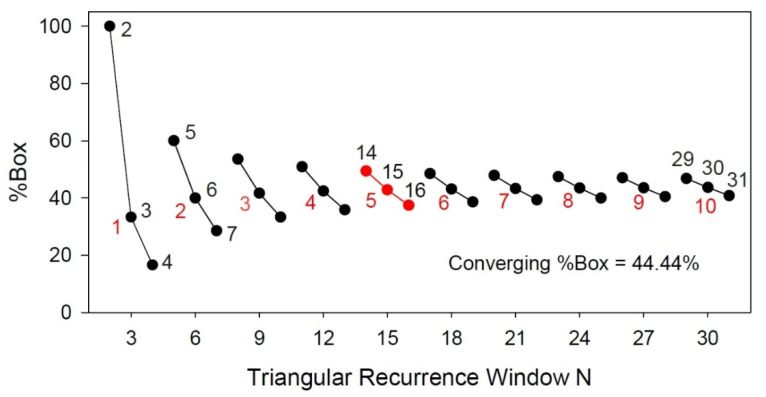
Ratio of tilted-box area to recurrence triangular area (%Box) in sets of 3 for increasing number of points in triangular recurrence window. Box sizes are shown in red integers. The triplet points (14-15-16) for the 5 × 5 box size as described in the text are also shown in red.

Clearly, the “square” tilted boxes can fit within 3threeincremental sizes of recurrence matrices. To compute the integer length of the box side one needs only to divide the number of points in the triangular recurrence window by 3. Thus, for our trio example: 14/3 = 4.667 (round up to 5); 15/3 = 5.000 (retain as 5); 16/3 = 5.333 (round down to 5). Now the question arises, what happens to %Box (area ratios) as the number of points in the recurrence window increases? This answer is shown graphically in Figure 5 for multiple sets of triplets. The 5 × 5 example is indicated in red. The slope of each trio decreases as the number of points increases. For example, for set 1001-1002-1003, the area ratios are 45.51%, 44.42% and 44.33%; for set 2000-2001-2002, the area ratios are 44.48%, 44.43% and 44.39%; for set 2999-3000-3001, the area ratios are 44.47%, 44.44% and 44.41%. In the limit for large set numbers, the area ratios converge on 44.44% for all trios. All this means is that the area of the new tilted box is approximately 44% of the area of the traditional recurrence triangle.

## 6. Logistic Equation

**Figure 6:**
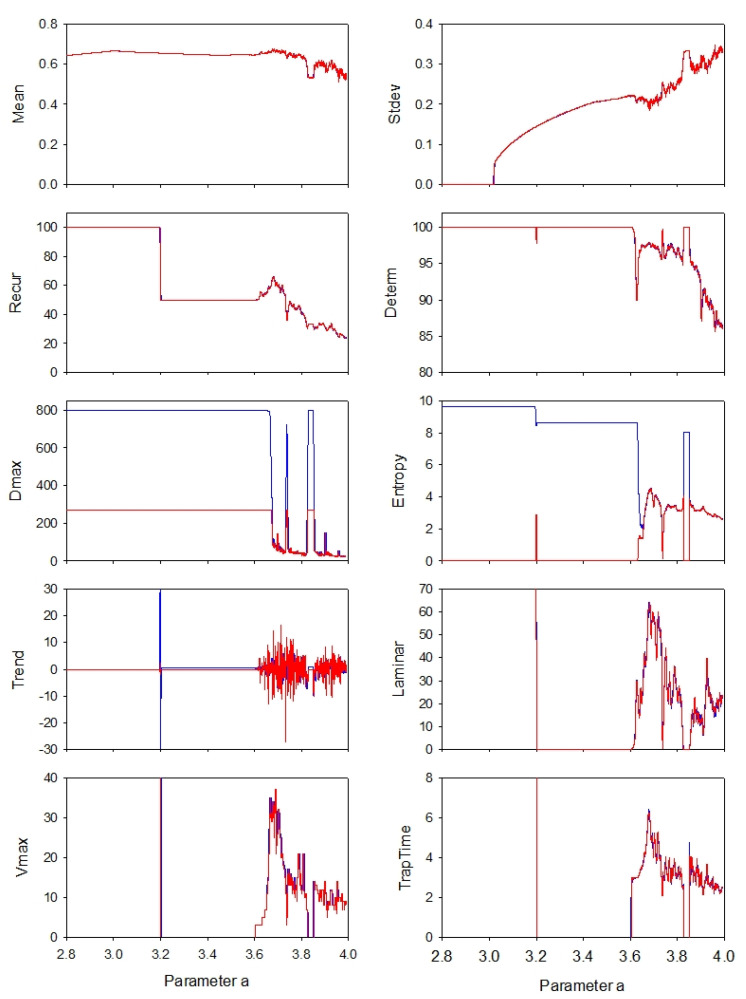
Recurrence variables of the logistic map computed by traditional RQA (blue lines) and boxed RQA (red lines). The logistic time series consisted of 120,001 points for a values ranging from 2.8 to 4.0. Each sliding window epoch consisted of 800 points which were shifted by 10 points (98.75% overlap) to yield 11,920 points per variable. Superimposed values are shown in red; divergent values are shown wherever blue appears. Parameter settings: DELAY = 1; EMBED = 3; NORM = Euclid; WINDOW = 800; SHIFT = 10; RESCALE = absolute; RADIUS = 1; LINE = 2.

At least three studies have examined the logistic map in terms of entropy over a range of control parameter a values [10,12,16] (Equation (7)). Each takes a different perspective on entropy calculations with pros and cons. The present study followed the procedures of the earlier study [10] in which parameter a was incremented on each iterated cycle in steps of 0.00001 from a = 2.8 to 4.0 yielding 120,001 points. This series of points was subjected to 800-point moving-window recurrence analyses using the traditional recurrence and boxed recurrence programs (program3 RQE and RQEB). The results for all eight recurrence variables are superimposed in Figure 6 with blue representing the traditional values and red indicating the boxed values. As shown by the tilted blue arrow in Figure 4, the mean and standard deviation values were computed from the middle third of time series points. Additionally, as shown by the horizontal red arrow in Figure 4, the boxed recurrence variables were shifted left by one-third of the window size (267 points = 800 points/3) to better align with the triangular recurrence variables.

X_n+1_ = a · X_n_ · (1 − X_n_) (7)

In most cases, the red traces overwrite the blue traces, proving excellent agreement with both approaches. However, there are three notable exceptions. First, the ENT values are widely divergent when the logistic system was in its fully periodic modes (period 1, period 2, period 4, period 8, etc.) over the approximate range of a = 2.8 to 3.6 with spikes just over a = 3.2. Additionally, during the period 3 window (a = 3.83) the 2 entropy values diverge.

Second, the DMAX values superimposed nicely, save during the sliding periodic windows. Because the window size was selected as 800 points, the traditional DMAX peaks at 799 diagonal points (just next to the LOI). However, the boxed window was only one-third the size of 800 which is why the boxed DMAX is clipped at 267 diagonal points.

## 7. Radius versus Embedding

**Figure 7:**
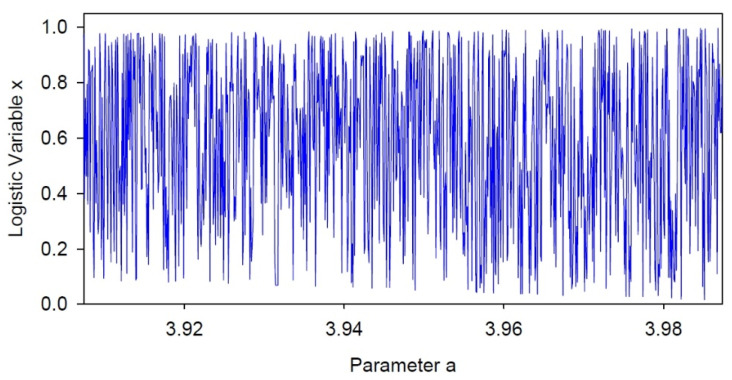
Fully chaotic mode of the logistic equation within an 800-point window (a = 3.9074 to 3.9873). The logistic x variable fluctuates wildly within the bounds of 0.0 (minimum) to 1.0 (maximum).

In RQA, one of the most difficult parameters to select is the radius. If the radius is too low, the number of recurrent points will be too sparse; if radius is too high, the number of recurrence points will be too dense. Indeed, when the radius equals or exceeds the maximum distance in the distance matrix, the recurrence plot will completely saturate (RECUR = near 100% and DETER = near 100% for LINE = 2). Taking advantage of this principle, it is possible to increment the radius and see at which point the entropy values for the triangular RQA and boxed RQA diverge. This was accomplished by using the new boxed RQA program RQSB. Secondly, these divergent points must be a function of the embedding dimension. To quantify these ideas, a single 800-point window was selected from the logistic map (a = 3.9074 to 3.9873) as shown in Figure 7. This window represents a purely chaotic window with no regular periodicities present. The signal looks stochastic, but because it is derived by iterating a nonlinear equation it is actually fully deterministic. This does not mean that the DETERM is 100%, however, because the various orbits are not equally close to one another. Increasing the radius, includes more and more orbits within the recurrence zone.

**Figure 8:**
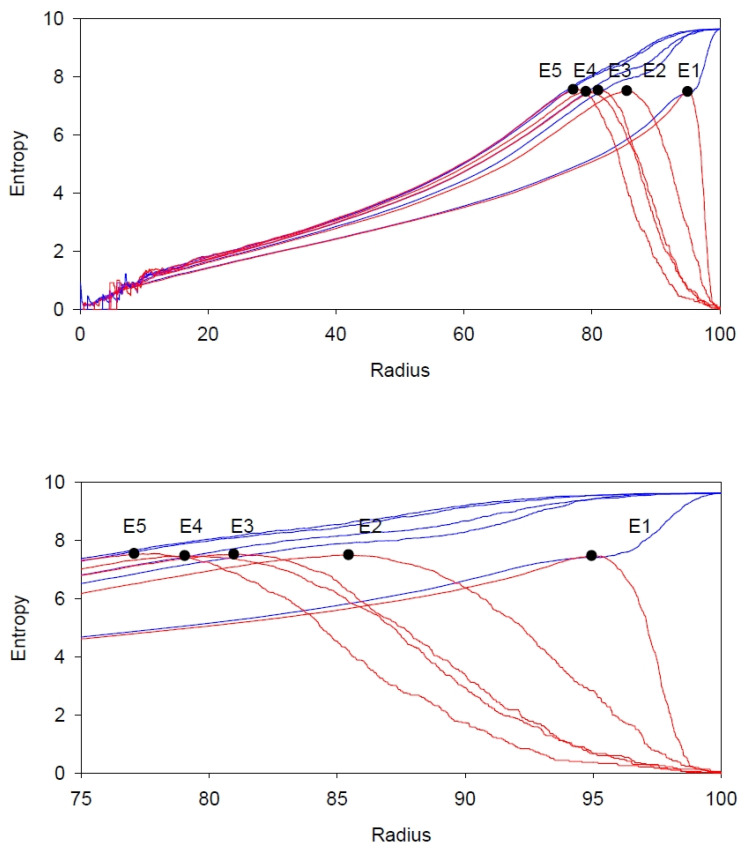
Line entropy values as a function of increasing radius values for five different embedding dimensions (E1, E2, E3, E4 and E5) for regular RQA (blue lines) and boxed RQA (red lines) computations. The black points indicate the radius value when the two entropy values diverge (turning points). The full range of radius values (top traces) are magnified for the upper quartile of radius values (lower traces) to show better discrimination of the parameter settings. Parameter settings: DELAY = 1; EMBED = 5; NORM = Euclid; WINDOW = 500; RESCALE = maximum distance; RADIUS = 10%; LINE = 2.

The computational results are shown in Figure 8, where traditional entropy calculations are displayed by the blue lines and the boxed entropy calculations are displayed by the red lines (programs RQS and RQSB). As can be seen, both entropy values track very closely until the density of the recurrence plots becomes too great at very high radius values. At these turning points the two entropy values begin to diverge as identified by the black dots in each trace. As the embedding dimension increases the turning point shifts to the left. For the five increasing embedding dimensions, the five decreasing radius values at the turning points are, respectively: RADIUS = 94.94%, 85.44%, 80.95%, 79.04% and 77.86%. At the various turning points, all entropy values, triangular and boxed, are bunched within a rather tight or narrow range (ENT = 7.438 to 7.913 bits/bin) albeit at high values. Additionally, as expected, when the radius value reaches 100% of the maximum distance, the two entropy values depart to their separate corners: regular entropies to their maxima of 9.64 bits/bin; boxed entropies to their minima of 0.00 bits/bin. What can be concluded from these results is that the triangular and boxed entropy values are so similar that the triangular RQA computations for entropy remain valid just as long as the systems under study are not fully periodic in the absence of noise.

## 8. Dow Jones Industrial Averages

As shown above, the noise-free sinewave signal presented above represents an extreme example of divergent entropy computations for verses triangular recurrences verses boxed recurrences (ENT = 3.907 vs. 0.000 bits/bin). Adding noise to the sinewave converges the two entropy values (ENT = 3.000 vs. 2.807 bits/bin). However, what happens to all eight recurrence variables when the recurrence area is constrained (masked) to the tilted box as compared against the standard recurrence triangular recurrence area?

**Figure 9:**
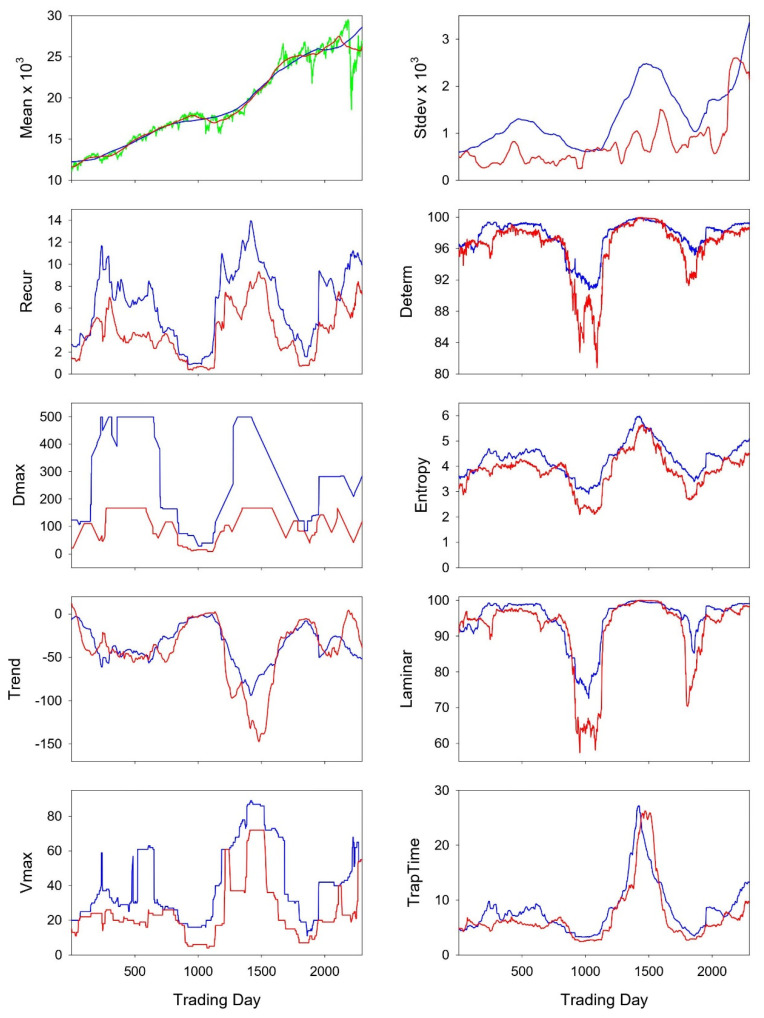
The mean values (left) and standard deviation values (right) are plotted for both the triangular area (blue) and tilted-box area (red). The means line up nicely, but there is less agreement for the standard deviation values. The raw Dow Jones scores are superimposed in the graph of means (green). Second, nonlinear descriptors of the Dow Jones scores are shown in the remaining eight panels (Figure 9). Each recurrence variable will be reviewed one by one, remembering that blue curves refer to triangular recurrences while red curves refer to tilted-box recurrences.

To answer this question, real-world data (noisy) were studied by both recurrence methods and the results were compared. The input time series consisted of downloaded scores of the Dow Jones Industrial Average for over a decade [17]. Recurrence quantifications were computed within a sliding window (programs RQE and RQEB) and the results are shown in Figure 9. Since the window size was 500 points, the length of the box side was 167 points (500/3) and %Box was 44.58% (see Equations (4)–(6)). Again the mean and standard deviation were computed for the middle third of the time series within each window, and the recurrence variables were left time shifted as explained in Figure 4.

1. RECUR: Recurrence rates for triangular and boxed recurrence areas are very comparable, not with exact value matching, but by their directional shifts while moving through the Dow Jones scores.
2. DETERM: Determinism scores are very comparable, quantitatively and qualitatively for the most part, save for dips (red) surrounding trading days 1000 and 2000.
3. DMAX: the longest line lengths with the moving window have minimal agreement. Both series are flat-topped around days 300 to 500 and days 1400 to 1500. The triangular measurement peaks at 499 points, but the boxed measurement peaks at 167 points because its area is lower by 44%.
4. ENTROPY: Despite some variability the two entropy traces track very nicely with each other. In one sense this is proof of concept that the presence of noise in the time series actually has entropy values converging.
5. TREND: This variable quantitates the paling of the recurrence plot away from the LOI. The two traces are almost superimposed save points surrounding trading day 1500.
6. LAMINAR: This variable reports the percentage of recurrent points that form vertical line structures. Qualitatively, the two curves match nicely. Quantitatively, the two curves also match nicely except for dips (red) near days 1000 and 1800.
7. VMAX: This variable is analogous to DMAX, but it measures the length of the longest vertical line. There is some qualitative agreement between both traces, but these results are more difficult to interpret.
8. TRAPTIME: This variable defines the average vertical line lengths which are rather identical for triangular and boxed recurrences.

## 9. Square Wave Pulse

**Figure 10:**
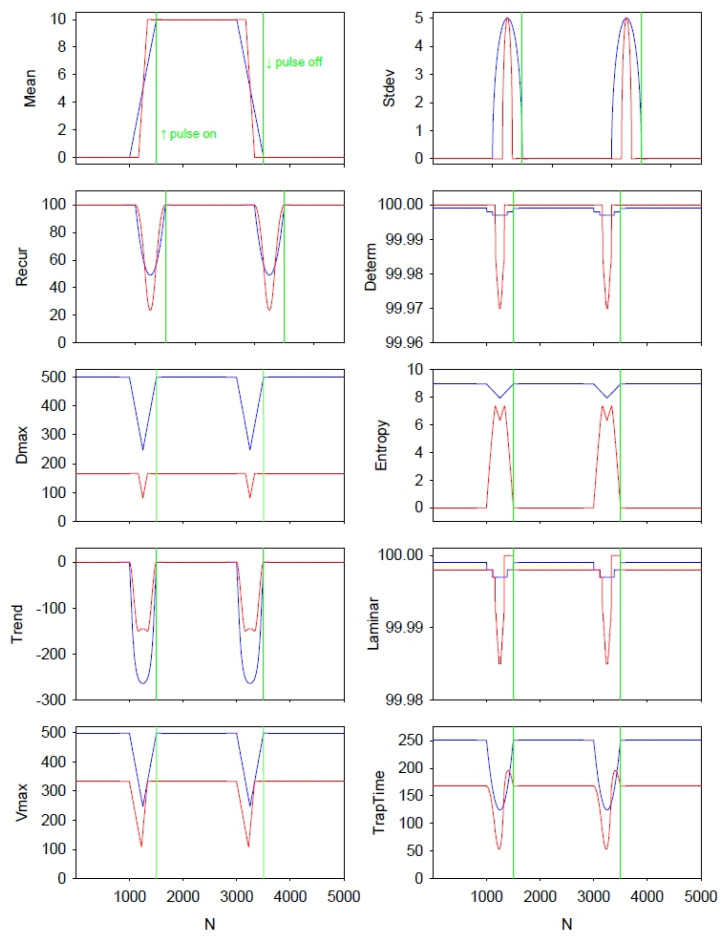
Comparison of traditional and boxed recurrences on a single square wave of 7500 points for 2 linear and 8 nonlinear recurrence variables. Triangular RQA computations (blue traces) and boxed RQA computations (red traces) are performed within a sliding window of 500 points offset by a single point between epochs for a total of 6977 epochs. However, only the first 5000 epochs are plotted. The onset of the rising square wave is indicated by the first vertical green line (pulse on); the end of the falling square wave is indicated by the second vertical green line (pulse off). Both time series were aligned to point 1 (see text). Parameter settings: DELAY = 1; EMBED = 5; NORM = Euclid; WINDOW = 500; SHIFT = 1; RESCALE = maximum distance; RADIUS = 10%; LINE = 2.

To better understand timing and amplitude relationships among all eight recurrence variables computed by the standard RQA versus the boxed RQA, the two methods were compared using a simple square wave input signal. This signal consisted of 7500 points in which the first 1500 points and last 4000 points were set to 0, but the middle 2000 points were set to 10. The first and second transitions were abrupt (pulse up: 0 to 10; pulse down: 10 to 0, respectively). Figure 10 presents the results of this square wave as processed by both the traditional RAQ and boxed RQA computations for all eight recurrence variables (programs RQE and RQEB). The sliding window approach was taken at the highest sensitivity (only 1 point between epochs) to allow for clear timing delineations. Again It should be noted that the boxed recurrences were aligned with the regular recurrences, plotting both time series from point 1. That is the boxed recurrences were plotted from point 1, not point 167 (or 500/3). Additionally, the double vertical green lines in each panel show the exact onset and offset of the square wave pulse.

The first observation is that width of the means and standard deviations are broader for the standard RQA than for the boxed RQA. This is because the tilted box spans fewer pulse points than the triangular space. Both means start from the same base (0.0) and rise to the same height (10.0), but the rise in the boxed mean begins later and has a greater slope. It can also be noted that the two standard deviations both reach identical maxima.

Second, prior to the onset and offset of the square pulse, the paired recurrence variables have peaks or nadirs that line up in time. Additionally, as is necessary, the interval between peaks or nadirs is exactly 2000 points which is the width of the square wave pulse.

Third, the contours and amplitudes of the paired recurrence variables are not the same. This is explained by the fact that the boxed recurrence area consists of only 55,611 pixels which is but 44.58% of the triangular recurrence area (124,750 pixels) (see Equations (4)–(6)).

Fourth, during the steady-state phases of the input signal (string of 0 s pre- and post-pulse; string of 10 s during the pulse), the regular entropy is pegged at 8.963 bits/bin (incorrect) whereas the boxed entropy is minimized to 0.000 bits/bin (correct). Only during the two transition phases do the two entropy values approach each other, but never meet. With the exception of these two entropy values and possibly the LAMINAR and TRAPTIME variables, the other five RQA variables exhibit similar directional changes during the two transition phases.

## 10. Discussion and Conclusions

Back in 2015, Marwan and Webber [18] discussed in detail the mathematical and computational foundations of recurrence quantifications. However, no mention was made about masking the recurrence matrix to modify the triangular border truncations of diagonal lines. Later, both of these authors independently posited a tilted rectangle [9] or tilted box (present paper) masking of the recurrence matrix such that long diagonal lines are clipped to identical lengths thereby minimizing the line entropy computations. It should be emphasized that both entropy calculations, triangular entropies and boxed entropies, are accurate, but have different meanings. In the traditional case, line-entropy values really describe the actual probability distributions of truncated lines. However, in the tilted box case, line-entropy values better correspond to the underlying dynamic itself. That is, low entropy values are related to simple (or random) systems, whereas high entropy values are usually correlated with more complex systems, but not necessarily always. As mentioned previously, Censi et al. [11], Eroglu et al. [12], Corso et al. [13] and Leonardi [14] all have thoughts about revised entropy computations. The question is not which method is the best, but rather, based upon the assumptions of each approach, how do the various entropy computations perform on systems of all sorts. This becomes an invitation for performing comparative studies applying these differing methodologies to representative systems of interest (beyond the scope of this paper).

For example, Leonardi [14] has a very nice summary description of the meaning of entropy, especially the information entropy of Shannon [8]. Information entropy values approaching maximum entropy (such as for random systems) contain high levels of information, are complex in nature, and unpredictable in principle. However, information entropy values approaching minimum entropy (such as for highly periodic systems) contain low levels of information, are simple in construct, and can be very predictable. This last statement is qualified for white-noise processes which present with very sort line structures which translate to very low entropy values as well. In short, the higher the entropy value, the more interesting is the system. Conversely, the lower the entropy value, the less interesting, even boring, is the system (excepting white noise). So for the sinewave, which is definitely periodic, very linear, and very repetitive (if you have seen one, you have seen them all), the entropy minimum of 0.000 bits/bin best describes the diagonal-line entropy. The tentative conclusion is that boxed recurrence entropies may have an advantage over triangular recurrence entropies. However, confirmation of such a claim must be verified by other correction schemes [9].

Many observations in this paper suggest that entropy values, if not most other recurrence variables, computed from triangular and boxed recurrences may not necessarily be that different after all. First, the addition of subtle noise to a pure sine wave collapses both entropies to nearly the same values insofar that long diagonal lines are disallowed (Figure 1, Figure 2 and Figure 3). Second, the logistic equation in its non-periodic and chaotic modes assigns very similar quantitative (overlapping) values to all recurrence variables (Figure 6). Third, both entropies track together for the chaotic state of the Logistic system until breaking points of separation occur only at very high radius values (Figure 8). Additionally, fourth, moving-window recurrence analyses of the Dow Jones Industrial Average data reveal correlations between triangular and boxed recurrence variables that have similar qualitative (REC, DET and LAM) or quantitative (ENT, TREND and TRAPTIME) profiles. With the exception of two poorly correlated variables (DMAX and VMAX), such patterns would lead to singular, not diverse, interpretations of the underlying dynamic (Figure 9).

As an aside, it will be appreciated that the two tilted maskings of the recurrence matrix are qualitatively similar for both Kramer and Marwan [9] and the present paper. What distinguishes the two approaches quantitatively, however, is that the area of the masked rectangular recurrence plot is a full 50% in the limit of the triangular matrix whereas the masking of the tilted box is only 44%. This 8% difference may constitute an advantage for the former insofar that it captures a greater proportion of recurrent points. This slight edge may be a fine tune difference between masked versus unmasked recurrence plots and quantifications. This hypothesis could be studied using various signals.

Finally, the moving-window recurrence analyses of the square wave pulse are provided basically for heuristic purposes alone (Figure 10). The input signal is very clean (noise-free) and two instantaneous jumps occur in the time series (pulse on and pulse off). One can visualize the unmasked triangular window and the masked boxed window both moving through the vertical lines. The responses in all eight recurrence variables reflect the two transitional state changes encountered. In addition, the effect of the left shift of the boxed recurrence variables can also be studied clearly. These plots can assist in learning how to read recurrence plot variables when using masked versus unmasked methodologies.

Perhaps the most surprising conclusion of this paper is that box masking of the recurrence matrix is not necessarily called for in most practical situations. Nevertheless, this study reveals that border truncation effects are real when dealing with long (even infinite) time series. If this observation can be verified for different systems it would mean that all previous studies relying on the traditional entropy calculations for systems that are even near periodic, the entropy values and other recurrence variables computed and reported are valid. A good example is one recent master’s thesis on which the conclusions relied heavily on traditional triangular entropy values to come to consistent and meaningful conclusions [19].

## Data Availability

The data reported in this paper can be requested directly from the author.

## Appendix A

The new software programs used in this paper, all written by the author, are bundled within the suite of RQA 2021 programs. These new programs include RQDB (recurrence display) RQEB (recurrence epoch), and RQES (recurrence scale) which were run to compute the boxed recurrences in this paper. They correspond to traditional triangular recurrence programs RQD, RQE and RQS. All RQA programs are embedded within a single ZIP file [20] which is easily downloaded at no cost. C-source codes will be made available to anyone for the asking.
